# ‘Mind-forg’d Manacles’: Virtual Experience and Innocent Publication

**DOI:** 10.1007/s11196-023-09971-4

**Published:** 2023-02-21

**Authors:** Francine Rochford

**Affiliations:** School of Law, La Trobe University Bundoora, Melbourne, Vic Australia

**Keywords:** Defamation, Publication, Facebook, Participatory governance, Semiotics

## Abstract

In *Fairfax Media Publications Pty Ltd v Voller* (‘*Voller*’) the Australian High Court held that media companies maintaining Facebook comment pages could be liable for the defamatory posts of commenters on those sites. The decision focussed entirely on whether, by maintaining the Facebook page, the companies had ‘published’ the statements of commenters. Hearings on other aspects of the tort litigation continue. This paper considers the implications of the tort of defamation on public participation on political will formation where, as is increasingly the case, the participation occurs virtually. Australian law has already tackled the law of defamation as a threat to freedom of political communication; *Voller* continues the jurisprudence by considering whether hosting an online forum for debate amounts to publication. The more recent High Court judgment in *Google LLC v Defteros* demonstrated the necessity of the law to align the ‘acts’ necessary to found legal action with the new environment of automated search engines. The troubled intersection of dematerialised practices of political and cultural discourse and jurisdictionally bound laws of defamation challenges participatory governance as tribes form and dissolve and shift between geographical interests.

Defamation in Australia is a tort of strict liability; and, absenting applicable defences, any participation in communication is sufficient to make that participant a publisher and a party to the defamation. The online environment stretches words across geographical and jurisdictional boundaries, but it also stretches and contorts concepts of fault and responsibility. Participatory digital cultural practices integrating users in the creation of cultural heritage simultaneously draw participants into transgressions, both cultural and legal, which are amplified by the medium. Questions of collective guilt, ‘shades’ of moral responsibility and disproportionality between blameworthiness and legal liability challenge laws formulated for the printing press but now deployed in the online environment. In this way the digitized participatory environment presents deep challenges to law and legal systems, which are chained to geography. This paper considers the concept of innocent publication in the context of the digitized participatory environment and the way in which the virtual experience is dissolving concepts of geographically defined jurisdictions.

In every cry of every Man,/ In every Infants cry of fear,

In every voice: in every ban, / The mind-forg’d manacles I hear [3].

## Introduction

Legal texts are ‘loaded with signs’ ([6]: 15). Legal publication is one of the early forms of record-keeping, acting as a repository and social memory which functions as an organising structure and a process of iterative knowledge creation. In the context of legal precedents as they operate in common law systems the insight that law is an ‘omnipresent progressing project’ ([6]: 18) is clearly observable. However, ‘a legal term only denotes in a particular temporal and spatial context’ ([7]: 167) – Blake’s ‘mind-forg’d manacles’, observed in language and law as limits to our understanding. Meaning is acquired in context and can change when context changes. The common law enables both conservatism and dynamism in the sense that a precedent must govern lower courts but may be distinguished as facts and context change. It is in the law’s adaptation to fundamental shifts in social structures, as in the rapid development of communication and action in cyberspace, that the conservative brake on social dynamism is most marked. In a semiotic reading of law’s unfolding response to new modes of communication, the law of defamation (libel and slander) is a useful starting point. The modern law of defamation as it applies to cyberspace engages issues of unsettled or shifting meaning of words, images, symbols and other communicative processes, the process of publication in non-corporeal form, the publication of matter without human agency and the movement of words and other symbols across jurisdictional and cultural contexts. It thus requires analysis of contemporaneously divergent meanings across cultural contexts mapped onto a framework of legal meaning created by self-referential reasoning. Added to this, in Australia the law of defamation challenges but must be read consistently with the process of political will formation as a foundation to the Constitutional democracy.

Semioticians distinguish between legal discourse in state-bound systems and legal discourse in a global system ([6]: 15). In this analysis we consider legal discourse in state-bound systems as it applies to regulate speech over the internet. Legal discourse in state-bound systems exercises different functions from legal discourse in global systems, but the speech to be regulated traverses multiple sites of regulation, raising both principled and pragmatic questions. The nature of the state is called into question, as traditional markers of state sovereignty are interrogated, reinterpreted or subverted. The question of communication agency is also reimagined, as algorithmic programs replace, and even manipulate, human action.

This chapter is a reflection on the decision in the Australian High Court in *Fairfax Media Publications Pty Ltd v Voller* [2021] HCA 27 (*Voller*). That case, in which the High Court was called upon to rule on the nature of a ‘publisher’ for the purposes of the law of defamation, raises questions about the nature of law in cyberspace. In the context of the digitalisation of many forms of communication, the capacity for communications to cross jurisdictional boundaries and to move between cultures, and the storage and reanimation of matter across temporal nodes, the simultaneous creation of multiple semiotic relationships challenges established legal understandings.

This analysis first briefly recounts the matter at issue in the High Court in *Voller*. It then considers the problem inherent in communications in cyberspace in a system of law which is geographically bounded, referencing the jurisprudence on property to interrogate the traditional physicality of jurisdictional limits. Noting those traditional markers of sovereignty that govern law’s applicability, the analysis then raises the question of how these markers are translated in cyberspace. It argues that the law of defamation, which must act consistently with the freedom of political communication implied into the text and structure of the Australian Constitution, is particularly vulnerable to the shifting context shaping communicative meaning.

## The Facts, the Issue and the Law

In *Voller* the plaintiff, Dylan Voller claimed that he had been defamed by various Australian media organisations. The organisations, Fairfax Media Publications Pty Ltd, Nationwide News Pty Limited and Australian News Channel Pty Ltd, were (and remain at the time of writing) publishers of newspapers, operators of television stations or both. They all maintained Facebook pages on which they posted content and they invited people to post comments. Voller claimed that some of these comments were defamatory. The matter was escalated to the High Court; not to finalise the proceedings, but rather to determine a matter of public and legal significance. The only matter to be resolved by the High Court was whether, for the purposes of the law of defamation, the news organisations were ‘publishers’ of the comments left by others on the Facebook pages. The High Court consisted of Chief Justice Kiefel and Justices Gageler, Keane, Gordon, Edelman, Steward and Gleeson. A 5:2 majority (Edelman and Steward JJ dissenting) held that the defendants had facilitated, encouraged and assisted in publication of comments and were, therefore, publishers for the purposes of the Australian tort of defamation.

The Australian tort of defamation was originally a creature of the common law and borrowed heavily from the English jurisdiction. The common law rules have been increasingly challenged by disproportionately large defamation compensation payments, highly technical rules that resulted in complex and extended proceedings, and inconsistency between defamation law and the freedom of political communication implied by the Australian Constitution. The implied freedom of political communication was developed and refined in a series of cases in the High Court, in particular: *Lange v Australian Broadcasting Corporation* (1997) 189 CLR 520; *Nationwide News Pty Ltd v Wills* (1992) 66 ALJR 658, *Australian Capital Television Pty Ltd v The Commonwealth [No 2]* (1992) 66 ALJR 695 and *McCloy v New South Wales* [2015] HCA 34. As the early cases established that the tort of defamation was capable of limiting the implied freedom, the common law has been substantially altered by legislation. The legislation is now moving towards uniformity in all Australian jurisdictions - national uniform defamation laws came into force in 2006, passed by each state jurisdiction based on a model uniform law developed by the Standing Committee of Attorneys-General. Inconsistencies remained between jurisdictions. Model Defamation Amendment Provisions 2020 were passed by Victoria, New South Wales, South Australia and Queensland and came into effect in 2021. However the statutes do not codify the law and much of the common law continues to apply.

The basic requirements of an action in defamation are that a ‘defamatory matter’ which ‘identifies’ the plaintiff is ‘published’. Each of these requirements are legally complex enquiries, built and honed over generations of precedent and now incorporated into legislation which borrows and builds upon precedent, but which also adds additional requirements. Whilst the High Court in *Voller* considered only the question of whether the defendants were ‘publishers’ within the meaning of the law of defamation, it is worth noting the complex semiotic relationships inherent in the construction of the ‘defamatory matter’. The task of the court in each case is to determine whether one or a number of ‘matters’ contained a defamatory imputation. The ‘matter’ need not be a word: it could be an image, gesture, effigy, signwriting in the sky, cartoon or caricature, song, photograph, painting or advertisement. The matter is defamatory by reference to ‘hypothetical referees’ – ‘Lord Selborne’s reasonable men … or Lord Atkin’s right thinking members of society generally or Lord Reid’s ordinary men not avid for scandal’ as described by Brennan J in *Reader’s Digest Services v Lamb* (1982) 150 CLR 500 (at 505). The imputation, or meaning of the words used, requires determination, and it is assumed that the hypothetical referees have a uniform view of the meaning of the language used. Once that has been determined the defamatory character of the imputation is evaluated by reference to the moral or social standards of those hypothetical referees. Again, as Brennan J noted in *Reader’s Digest Services v Lamb* (at 505) those referees are taken to share the moral or social standard. That these standards vary temporally is readily apparent. The cultural divergence between geographical areas, or spatial nodes ([7]: 177), is similarly marked. Where publication occurs simultaneously over disparate cultures, as in the case of publication using the internet, the task of ascertaining meaning and determining alignment with the standards of hypothetical referees becomes wickedly difficult. In fact, the task of the court is corralled by its jurisdiction, but from the perspective of the ‘publisher’ on the internet the standard, or defamatory connotation, of matter may shift between the original poster in one cultural context and the ‘publication’ in another. Facebook, and the internet generally, moves across multiple cultural contexts simultaneously, and can even traverse temporal contexts. Matter which is uncontroversial in one spatial or temporal context can change when transported to another.

## Law and Geography

The ‘gist’ of the civil law of defamation is whether the defamatory matter has been published, and publication traditionally fixed the matter within a geographical site – the jurisdiction of the Court is bounded by territoriality in a physical sense. The question of publication is satisfied by communicating to a third person, but typically other strategic considerations (including the harm caused by the defamatory assertion, and consequently the availability of compensation) will dissuade an aggrieved person from bringing an action unless publication has been widespread. However, the tort of defamation protects reputation, and in many cases a person’s reputation would be geographically limited. The growth of the internet has changed this dynamic, so that not only can a person more easily develop a reputation across many jurisdictions, that reputation can also be readily damaged across many jurisdictions. In *Dow Jones v Gutnick* [2002] HCA 56; 210 CLR 575 the High Court resolved a jurisdictional dispute brought by Dow Jones seeking the relatively safe haven of New Jersey law, on which the online magazine article in dispute was stored. Dow Jones argued that the article was uploaded from web servers every time an internet user requested a copy of the article, so New Jersey was the appropriate forum. The High Court held that the state of Victoria was the appropriate forum, as the place at which the plaintiff’s reputation was damaged. Accordingly, the law of Victoria would govern the issue. The jurisdictional issues are still contentious, and forum shopping has the potential to undermine territorial jurisdiction in this field. Adjudicative jurisdiction can readily be attracted, piercing the territorial borders of state power, obviating the need for the physical presence and challenging fundamental aspects of originating proceedings such as serving process.

The spatial aspects of systems of justice align with geo-political boundaries and systems of enforcement, creating concrete and now assumed jurisprudential infrastructure. The law as an organizer of social relationships operates within the boundaries of its jurisdiction. Jurisdictional markers apply upon colonisation or revolution and have traditionally involved physical engagement with the territory to construct an excess of ‘floating signifiers’ [15]. These signifiers reaffirm the presumptions of physical jurisdiction. The ‘planting’ ([25]: 637)) of the flag symbolises the rule of the coloniser – the flag signifies sovereignty, whatever later meanings develop ([21]: 233). Flags carry ‘symbolic weight woven into the fabric … that imbues them with the deep meaning that they carry for so many across the world ([22]: 356). Reports of the foundation of the Australian colony indicate other cultural markers: the Governor and officers ‘drank toasts to the health of the King and the Royal Family and the success of the new Colony’ ([25]: 637). Marines fired the *feu de joie*, and all gave three cheers ([25]: 637). This process is part of an elaborate construction of artefacts to align with then established legal signifiers [10], thus performatively asserting sovereignty. ‘The display of the flag and the demonstration under it were intended as a reassertion and a making good of the title of the British Crown to the territory of which Cook had already formally taken possession in the name of the King’ ([25]: 637). The feu de joie, the ‘running fire’ deployed in celebration, is a militaristic token of the landmark occasion.

Along with sovereignty, the performance also asserted the ‘birthright’ of the settlers – the law of England – and reiterates the process by which a particular law is attached to a particular geographical site. Latham describes the effect: ‘As soon as the original settlers had reached the colony, their invisible and inescapable cargo of English law fell from their shoulders and attached itself to the soil on which they stood. Their personal law became the territorial law of the Colony’ ([13]:517). The soil of the colony was heavily imbued with meaning in English law and vested with cultural meaning associated with industry and plenty. The law ‘took root’ and bore fruit ([25]:636). The infrastructural presumptions of Australian law are thus based on a history of English land law. In English feudal terms sovereignty over the land was enmeshed in the legal device of ownership, traditionally marked by the physical act of handing over soil or dirt. In early English land law the transfer of land was accompanied by rituals referencing religion, the role of the lord in protecting the land, and the physical land itself. In *Manton v Parabolic Pty Ltd* (1985) 2 NSWLR 361 Young J described the ceremonies attached to the act of transfer of land – livery of seisin. As Young J describes in *Manton v Parabolic* (at 367).

originally the two parties to a conveyance attended on the land. The vendor removed his battle glove with which he had defended the land and vested the purchaser with it. It is, of course, from this ceremony that we get the words “vesting in possession” and the like. The vendor then took his knife and dug up a sod and lifted it up and handed it to the purchaser. This lifting up and handing over is the livery. The vendor then handed the purchaser the knife which was usually broken or twisted into a unique shape as a memorial of the transaction. The vendor then publicly quite [sic] the land and usually threw to the purchaser a wand or rod or festuca. No-one really knows exactly what the festuca was, but …there is no doubt it had a great contractual efficacy. The parties then repaired to the Church where the knife was usually laid up on the altar.

The ceremonial processes of land transfer, like the militaristic ceremonies of occupation, clothed law in ceremony to affirm and reaffirm the rights of ownership. In this way, however, law was also manifesting its own boundaries – the idea of law was manacled to the physical land. In English law, land ownership was first practically then symbolically aligned with power and the capacity to defend the land, and just as the individual conveyance referenced the requirement to fight, that obligation was manifest in the feudal system itself. In *Mabo v Queensland* (1992) 66 ALJR 408 (at 483) Toohey J noted that.

In considering the consequences of the annexation of the Islands, the distinction between sovereignty and title to or rights in the land is crucial. The distinction was blurred in English law because the sovereignty of the Crown over England derived from the feudal notion that the King owned the land of that country. It was ownership of the land that produced the theory of tenures, of obligations owed to the Crown in return for an estate in land. The position of the Crown as the ultimate owner of land, the holder of the radical title, has persisted.

At European settlement of the island continent now associated with the name ‘Australia’, measuring, mapping or remapping of borders became a cartographic discourse. The borders of the map create frontiers, and within borders property rights are established and mapped, creating ‘new interpretive references …to inscribe a new order upon the land.’ The cadastral map becomes ‘an established, if not axiomatic, adjunct to effective government monitoring and control of land’ ([12]:xvii). The naming of the Australian landscape by European settlers projected both reaching desire and imagined futures: ‘metaphors, desires for good pastoral land or permanent water projected onto the landscape’ ([24]:65). Material manifestations of sovereignty accompanied the progressive colonisation of the Australian continent, and those who understood the asserted meaning could arm themselves with the legal accoutrements to participation in the state. Thus, the state encouraged settlement for agricultural purposes by enabling selection of land by marking boundaries, clearing and fencing across one dimension. Similarly, licensing of prospecting encouraged miners to stake and work claims on Crown Land according to the requirements of the various state Mining Acts.

The meaning of the symbols of colonisation and governance have changed over time. Some have been reimagined - the physicality of the processes of land ownership and conveyance were mirrored in the evocative photograph of Gough Whitlam pouring sand into the hand of Vincent Lingiari to symbolise the provision of title [2]. Gurindji man Lingiari had been the leader of the 1966 walkout of Aboriginal workers from Wave Hill cattle station in the Northern Territory, which eventually resulted in the *Aboriginal Land Rights (Northern Territory) Act 1976* (Cth). The association between other symbols and their meaning has been disrupted or radically shifted. The common law has marked this shift by remapping and rearranging ‘legal icons’ [24]. Thus, the assertion of sovereignty inherent in planting the flag has been subverted by the creation and use of a new flag, created by an individual as a symbol of protest [22] (although the copyright to the flag was obtained by the Commonwealth of Australia and it is recognised in s 5 of the *Flags Act 1953* (Cth)).

The accoutrements of sovereignty are similarly apparent in the processes of determinative justice. Images of blinded Justitia [23], the remnants of colonialism in the coat of arms under which justice, metaphorically and literally, sits [16]; and the symbolism inherent in the incorporation of new state symbols by executive order in modern courts [16]. The architecture of the courtroom, separating legal actors, managing contact and sight-lines and governing movement, also has psychological effects. The semiotics of space govern the physical location of courts and the internal environment of the courtroom [15]. Conversely, recent reframing of court architecture to be sensitive to the manifestations of oppression inherent in European legal frameworks, and to incorporate ‘Aboriginal architecture’ [18] into design principles, may shift the language of experienced law. Similarly, the shift to virtual trials during lockdowns necessitated by the COVID-19 pandemic necessitated a rapid reimagining of the processes of determinative justice. The courtroom as a ‘physical site of justice’, open to the public and thus both symbolising the participation of the public in justice and acting as a brake on power, was replaced by ‘virtual hearings’. ‘Courtrooms … aim to create “‘an aura,’ a mystique of authenticity and legitimacy.” When proceedings are forced onto Zoom, Webex, or other virtual platforms, much if not all of that mystique or aura is likely to be stripped away’ ([1]: 1281; citing [8]:68)).

State marks of sovereignty over the geographical site to be governed thus shift and change in the wake of new ways of knowing or experiencing reality. Socio-economic and political fractures demand reinvention of the legal incidents of sovereignty, thus demonstrating that law is a ‘process of *knowledge of events, reality, being’* ([6]:18). The ‘self-constitution’ ([6]:18) of law, however, is more fundamentally challenged in its attempts to regulate non-grounded activities – the movement of words and incorporeal assets in cyberspace. The unfolding realities in the cyber realm are challenging to the law, which cannot control its own meaning and logic in relation to this domain. It is vulnerable to hegemony as state signifiers are diluted and dissolved by potent external signifiers. The deliberate meaning-making and re-making observable in the history of the Australian colony now interplays with meaning-making by both state and non-state actors, so that narratives germinate and spread regardless of physical territory.

## State, Sovereignty and Cyberspace

Translation of practices and symbols creating boundaries in sovereignty to cyberspace is problematic. The borderlessness of the Internet was judicially noted in *Dow Jones v Gutnick* [2002] HCA 56; 210 CLR 575 by Justice Kirby, who, citing case law in several jurisdictions, stated that ‘[t]he history of the Internet, its ubiquity, universality and utility have been described in the reasons of many courts in the United Kingdom [9], the United States [26], Canada [4], Australia [17] and elsewhere [14].’ To deal with the implications of borderless publication proxies such as sites of upload or download, residency of plaintiff or defendant, can create the geographic markers to jurisdiction.

In semiotic terms the law of defamation translated to cyberspace produces peculiar problems. Clearly, the ‘legi-signs’ [19], ‘the specific signs in words of law, legal doctrine or general jurisprudence’ ([6]:16) are discoverable and translatable. The relation between the legi-sign and the object, or even the interpretant, will shift between jurisdictional boundaries, language and cultural contexts. ‘The embeddedness of text, in itself and within the cultural milieu in which it is invoked, and internal to the individuals who undertake that reading, complicates sign, symbol and interpretant in an ever-evolving change of character with a change of position’ ([5]:vii). Whilst the genius of law lies in its capacity to fix the meaning of legi-signs in contemporary context and track changes in meaning by meticulous record-keeping, the object of the legi-sign and the interpretant are fluid temporally and across jurisdictional boundaries. Releasing the grounded referents and symbols of law into cyberspace to work across and between boundaries deeply challenges established correspondences, enabling the contention that ‘all meaning is transitory and merely an opening to something else’ ([5]: ix). The ‘mind-forg’d manacles’ of legal thought, the infrastructure of jurisdiction and the fine-grained analysis of precedent, are eroded and start to fall apart.

Ascertaining the defamatory meaning of a statement, gesture, image or other matter requires an often highly detailed analysis of the imputation – the meaning of the words or other matter, then the defamatory character of that imputation. In *Reader’s Digest Services v Lamb* (1982) 150 CLR 500, Justice Brennan noted (at 505)

Whether the alleged libel is established depends upon the understanding of the hypothetical referees who are taken to have a uniform view of the meaning of the language used, and upon the standards, moral or social, by which they evaluate the imputation they understand to have been made. They are taken to share a moral or social standard by which to judge the defamatory character of that imputation..

By assuming the uniform understanding of the hypothetical referee, the interpretant, the court assumes homogeneity across linguistic and cultural contexts. In the context of the internet this assumption is famously problematic. In practical terms, as the plurality noted in *Dow Jones v Gutnick * [2002] HCA 56; 210 CLR 575 (Gleeson CJ, McHugh, Gummow and Hayne JJ) ‘a publisher [is] forced to consider every article it publishes on the World Wide Web against the defamation laws of every country from Afghanistan to Zimbabwe’ (at paragraph 54). Whilst this is no doubt a boon to employment in pre-publication legal vetting, it constitutes a burden on freedom of communication.

It is trite and unarguable that speech is necessarily limited. There are many examples of speech acts which may attract criminal or civil sanction - incitements to criminal behaviour (as in *Michael Brown v The Members of the Classification Review Board of the Office of Film and Literature Classification* [1997] 474 FCA (6 June 1997) and *Michael Brown v Members of the Classification Review Board of the Office of Film and Literature* [1998] 319 FCA (24 March 1998)), hate speech (as in *Toben v Jones* [2002] FCAFC 158) and misrepresentations and misleading and deceptive statements are immediately brought to mind. In redesigning the law of defamation to comply with the implied freedom of political communication it was accepted that the law of defamation was a *burden* on freedom of political communication. The question was whether it was an *impermissible* burden. Impermissibility is not measured by politeness, rationality or deference to authority: in *Coleman v Power* (2004) 220 CLR 1 the High Court considered whether a Queensland vagrancy law was consistent with the implied freedom of political communication in prohibiting ‘insulting’ words. Whilst Heydon J suggested that insults did not form part of the ‘search for truth’ in political communications. Justice Kirby disagreed, noting that civility did not describe the Australian political system. He said (at [239]) that

One might wish for more rationality, less superficiality, diminished invective and increased logic and persuasion in political discourse. But those of that view must find another homeland. From its earliest history, Australian politics has regularly included insult and emotion, calumny and invective, in its armoury of persuasion. They are part and parcel of the struggle of ideas. Anyone in doubt should listen for an hour or two to the broadcasts that bring debates of the Federal Parliament to the living rooms of the nation. This is the way present and potential elected representatives have long campaigned in Australia for the votes of constituents and the support of their policies. It is unlikely to change. By protecting from legislative burdens governmental and political communications in Australia, the Constitution addresses the nation’s representative government as it is practised. It does not protect only the whispered civilities of intellectual discourse..

The High Court held in *Lange v Australian Broadcasting Corporation* (1997) 189 CLR 520, 567–568 that the law (in this case the common law of defamation) must be ‘reasonably appropriate and adapted to serve a legitimate end the fulfilment of which is compatible with the maintenance of the constitutionally prescribed system of representative and responsible government’. Defamation law has a legitimate end – the protection of the reputation of the individual – but the parameters of the law of defamation must be governed to ensure that it does not impermissibly intrude on the protected freedom. The compatibility of the tort of defamation with the freedom of political communication implied into the Australian Constitution lay in the defences to the defamation action. This was arguable where the communication was about government or political matters but could be defeated if the defendant’s conduct was unreasonable or actuated by malice. Defamation legislation has subsequently incorporated this: see for instance s 30 of the *Defamation Act 2005* (Vic).

There is a legitimate question about how ‘political’ communication has to be in order to fall within this area of Constitutional immunity. If ‘the personal is political’ for feminist scholars [20], the same could be said about any situation in which structural power is exerted in a manner which disempowers, limits or silences. Moreover, as the reach of traditional ‘politics’ is extended by a suite of communicative mechanisms largely facilitated by the internet, the political also may be said to have become personal. There is also a real question whether the ‘political’ is limited by traditional state sovereignty and its manifestations. The potentially disproportionate power of commercial organisations could be construed to be exertions of political power; an issue confronted by statutory reform. The uniform Defamation Acts prevent most for-profit corporations suing in defamation (for instance in s 9(1) of the *Defamation Act 2005* (Vic)). The dissolving of boundaries between personal and political has consequences: in permitting multiple assemblages of sign and meaning the burden on the interlocutor to manage legal risk may result in overly-nice, attenuated discourse. Theoretically at least this may impact more resoundingly on those with lesser facility in language – the young, the uneducated, or those whose work and lives are mediated by uncultured language. Political discourse in Australia is rarely ‘an intellectual salon where civility always (or usually) prevails)’, as Kirby J noted in *Coleman v Power* (2004) 220 CLR 1, and it is problematic to exclude communication characterised by Heydon J as ‘vices of intolerance rather than the virtues of tolerance’ in the same case.

Unfortunately, *Voller* did not test the boundaries of the question of the political. The High Court proceedings focussed entirely on the question of publication. The shifting ground of this concept was described by Gaegler and Gordon JJ (at [86]):

The advent of the Internet has resulted in a “disaggregation” of the process of publication and has facilitated a shift from “one-to-many” publication to “many to-many” publication. That technological and sociological development has not been shown to warrant a relaxation of the strictness of the common law rule [of publication].

Instead, *Voller* confirmed that enabling the display of third-party posts on an internet forum constituted publication of the resulting posts. A media outlet maintaining a Facebook page on which posts were made as was the case in *Voller*, bears legal responsibility if those posts are defamatory, regardless of whether the outlet was aware of the comment, and even though, as was the case at the time, Facebook did not allow the comments function to be disabled. In *Barilaro v Google LLC* [2022] FCA 650 the Federal Court compounded the vulnerability of the facilitator of an internet forum by holding that a platform which becomes aware of a defamatory comment and fails to remove it is also a publisher for the purposes of the law of defamation. In *Google LLC v Defteros* [2022] HCA 27 the High Court revisited the question of publication in the context of the provision of hyperlinks to defamatory articles. It held that the provisions of a hyperlink to a news article which contained defamatory material did not constitute publication, but that by providing a link to the relevant article the search engine Google did not contribute ‘in any extent’ to the publication of the defamation.

The precedents set are not restricted to the media, or even to those using Facebook as a marketing tool. A bill to obviate the effects of *Voller* (the Social Media (Anti-trolling) Bill 2022 (Cth)) was introduced in February 2022. In the second reading speech the Minister for Communications, Urban Infrastructure, Cities and the Arts stated that

The government is … concerned that the liability risks made clear by Voller may have a chilling effect on free speech—as page owners may censor comments or disable functionalities due to a fear of being held liable for content that they did not post. In some cases, the Voller decision may have contributed to decisions to limit the ability for the general public to interact with news and current events. We saw this, for example, when the US news network CNN blocked access to its Facebook pages in Australia after the Voller decision was handed down.

The Bill lapsed at dissolution of Parliament in April 2022.

Facebook (rebranded as Meta in 2021 to reinforce and market itself on the ubiquity of the ‘metaverse’) had positioned itself against restrictions proposed in Federal Government proposals to force the company, and others, to pay for Australian content. Demonstrating the market power wielded by the company and others, such as Google, Facebook’s actions reiterate the shifting power relations between state and commercial participants. As part of the bargaining around the proposed Code Facebook altered its preferences to enable users to ‘turn off’ comments on Facebook pages in response to the decision in *Voller.*

## Conclusion

The digitization of communication has enabled rapid disintegration of the limits of speech and has challenged the markers of jurisdictional sovereignty. Simultaneously it has increased the range and capacity for political discussion, potentially marking real participation in the political will formation said to be a necessity for representative and responsible government. In parsing the limits of political discussion, recent cases based in defamation law have continued the task of answering challenges to traditional ideas of publication, utilising analogies anchored in common law. Courts exercising jurisdiction based on state sovereignty have also had to acknowledge the shift from traditional markers of jurisdiction, and a rapidly expanding set of cases continue to challenge assertions of jurisdiction. For instance, the High Court has recently signalled that it is prepared to hear an appeal by Meta from the decision in *Facebook Inc v Australian Information Commissioner* [2022] FCAFC 9 on the jurisdictional issue: Facebook Inc v Australian Information Commissioner & Anor S28/2022 [2022] HCATrans 157, involving the collection of information from Facebook by Cambridge Analytica. At the time of writing the appeal had not been concluded. 

Other cases, such as *Trkulja v Google LLC* (2018) 263 CLR 149, have challenged notions of agency in questions of publication by pleading that algorithmic processes autopredicting terms and matching search terms with images and texts amount to defamation. In legal semiotics the law of defamation carried by incorporeal systems across cultural boundaries promises fruitful analysis. In this way, the application of legi-signs in cyberspace is a continuation of an evolution in semiotics, for ‘nothing is more semiotically driven than the deconstruction of the edifices within which semiotics may be encased, ossified, within a theory that both confines and entombs it in space and time’ ([5] (ix)).

In Blake’s *London* the poet refers to ‘Mind-forged manacles’ – a term invoking the invisible but constraining limits to the human imagination. Gramsci’s reference to ‘hegemony’ expresses the same concern [11]. The capacity of language itself to mediate the exercise of hegemony across states is, with the development of the internet increased; but novel forums for the discussion of political ideas are created. At the same time, mind-forged notions of states, sovereignty, and jurisdiction are thrown off, or out.
